# The accuracy and precision of acetabular implant measurements from CT imaging

**DOI:** 10.3389/fbioe.2023.1150061

**Published:** 2023-04-28

**Authors:** Johann Henckel, Angelika Ramesh, Harry Hothi, Robin Richards, Anna Di Laura, Alister Hart

**Affiliations:** ^1^ Royal National Orthopaedic Hospital NHS Trust, Stanmore, United Kingdom; ^2^ Department of Mechanical Engineering, University College London, London, United Kingdom; ^3^ Institute of Orthopaedics and Musculoskeletal Science, University College London, London, United Kingdom

**Keywords:** 3D-CT, inclination, version, acetabular component position, coordinate measuring arm, cup orientation

## Abstract

The placement of acetabular implant components determines the short- and long-term outcomes of total hip replacement (THR) and a number of tools have been developed to assist the surgeon in achieving cup orientation to match the surgical plan. However, the accuracy and precision of 3D-CT for the measurement of acetabular component position and orientation is yet to be established. To investigate this, we compared measurements of cobalt chrome acetabular components implanted into 2 different bony pelvic models between a coordinate measuring Faro arm and 3 different low dose CT images, including 3D-CT, 2D anterior pelvic plane (APP) referenced CT and 2D scanner referenced (SR) CT. Intra-observer differences were assessed using the Intraclass correlation coefficient (ICC). The effect of imaging the pelvis positioned in 3 different orientations within the CT scanner was also assessed. The measured parameters were the angles of inclination and version. 3D-CT measurements were found to closely match the “true values” of the component position measurements, compared with the 2D-CT methods. ICC analysis also showed good agreement between the coordinate measuring arm (CMA) and 3D-CT but poor agreement between the 2D SR method, in the results from two observers. When using the coordinate system of the CT scanner, the measurements consistently produced the greatest error; this method yielded values up to 34° different from the reference digitising arm. However, the difference between the true inclination and version angles and those measured from 3D APP CT was below half a degree in all cases. We concluded that low radiation dose 3D-CT is a validated reference standard for the measurement of acetabular cup orientation.

## 1 Introduction

The last decade has witnessed a substantial increase in the total number of hip replacements performed annually worldwide, with concurrent advancements in the surgical approach and technological tools used in the field. The use of robotic technology, computer-based navigation systems, patient-specific instrumentation (PSI) and custom implants for the placement of acetabular components all promise improved accuracy and superior reproducibility (Henckel et al., 2018; Krämer et al., 2018; Fontalis et al., 2021). If these are to be implemented on a wider scale, it is crucial to evaluate their accuracy using metrics such as inclination and version, which are frequently used to quantify acetabular cup position (Spencer-Gardner et al., 2016; Fontalis et al., 2021). Post-operative hip prosthesis measurements allow orthopaedic surgeons and companies to assess the achieved component position and orientation relative to the intended surgical plan. This is important to make robust assessment of new enabling technologies in surgery.

Measurement of hip implant positioning using 3D-CT has become the gold standard approach (Kaiser et al., 2021). Numerous studies have investigated the orientation of hip implants from post-operative CT scans and planar radiographs (Davda et al., 2015; Ma et al., 2022). However, the presence of metal artifacts and identification of anatomical landmarks on the reconstructed 3D pelvic model can introduce errors in the measurements of the implant position (Brownlie et al., 2020). These potential errors have not yet been quantified.

The novelty of this study includes the following.

• This is the first study quantifying the errors associated with CT measurements of acetabular cup orientation, from different coordinate systems, using artificial pelvic bone models. Accurate measurements can help evaluate the use of computer-aided systems which claim a 1–4° accuracy for cup alignment (Elson et al., 2015).
• Comparison of measurements taken from slices of the same CT scan before and after orienting the image using specialized software is a task which has not yet been undertaken.

The aim of our study was to quantify the accuracy and precision of 3D- and 2D-CT measurements of acetabular cup position for total hip arthroplasty (THA) outcome assessment. This will be achieved by evaluating and comparing the inclination and version of implanted acetabular cups using a) a coordinate measuring arm (CMA), b) 3D anterior pelvic (APP) referenced CT, c) 2D APP referenced CT and d) 2D scanner referenced (SR) CT. The effects of changes in pelvic orientation and inter- and intra-observer error on the measurements are also explored.

The four different methods adopted for measuring the cup inclination and version are outlined in this paper. This includes the identification and alignment to the APP. The results from the CT methods will then be compared to the reference standard in order to define the accuracy of each approach.

## 2 Materials and methods

**FIGURE 1:**
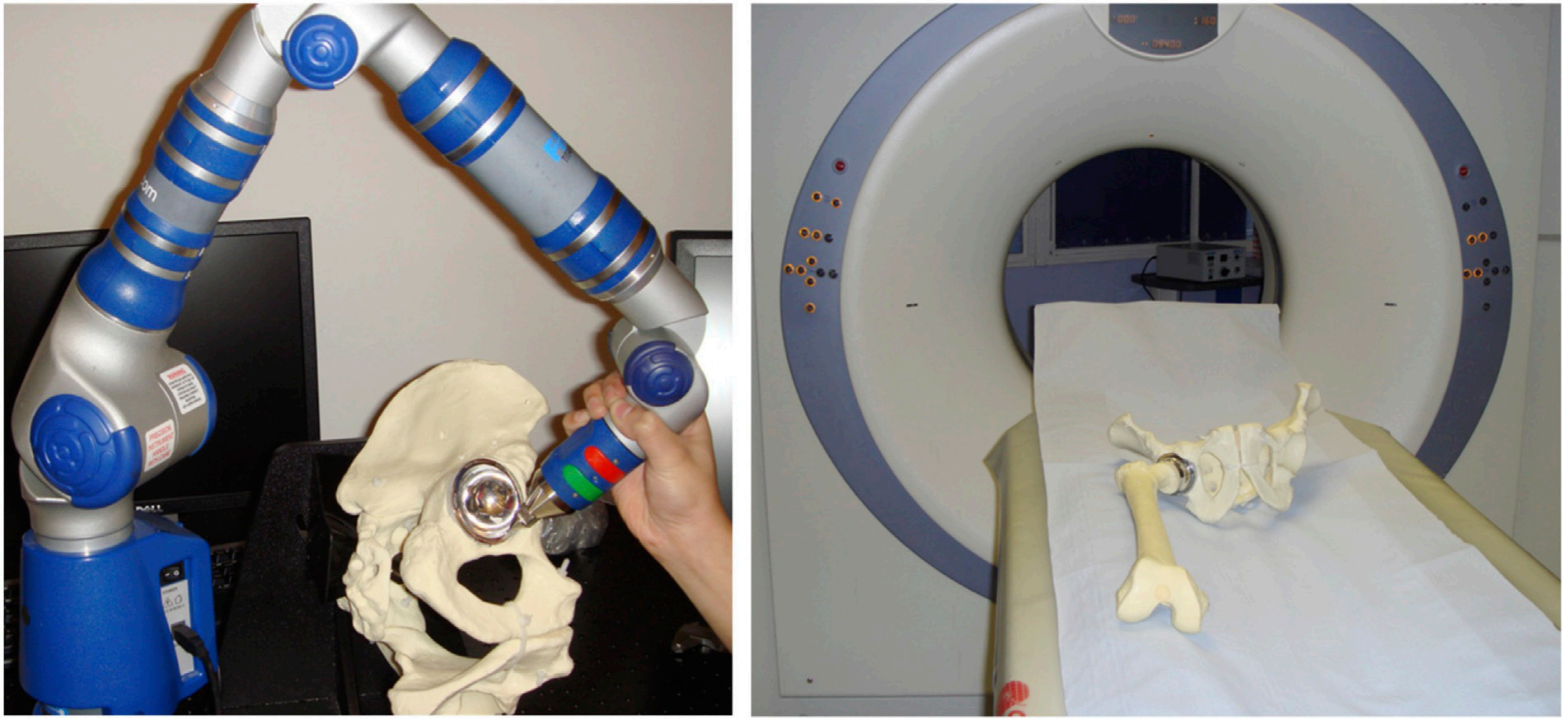
(Left) The orientation of the metal acetabular cup implanted in the pelvic Sawbone model being measured by the digitising arm. (Right) The model placed on the CT scanner table with the fiducials (hexagonal fully threaded titanium screws) placed on landmarks for robust landmarking.

Two pelvic artificial bone models (Sawbone Pelvis - Foam Cortical SKU:1301-1) were implanted with metal acetabular components (Figure 1). The pelvic height (HP) was 150 mm, the interspinous distance (ISD -distance between the two most prominent points of the anterior superior iliac spine (ASIS)) was 250 mm and the intercristal distance (ICD - distance between the most prominent points of the iliac crest) of the model was 270 mm (Musielak et al., 2019). The acetabular size was chosen by a senior orthopaedic surgeon to fit the Sawbone pelvic model used in this study.

Identification of both ASIS and pubic tubercles was made by placing the anterior face of the pelvis onto a flat surface covered with graphite to reveal the 3 prominences which define the APP. 5 mm fully threaded hexagonal titanium screws serving as “fiducial markers” were securely fixed into these 3 points, allowing for robust landmarking on the CT images without the associated imaging artefact, resulting from the use of metal implants. The two pelvic models were prepared using conventional tools to ream the acetabular socket to the size of the prosthetic cup. The cup was securely implanted in an orientation deemed suitable for the pelvis (approximately 45° of inclination and 20° of anteversion). A press-fit fixation was achieved and thoroughly assessed to ensure that there was no movement of the component relative to the bone.

### 2.1 Measurement of cup position using the coordinate measuring arm

The “post-op” pelvises were rigidly secured to a pelvic clamp. A digitising arm (reference standard, Gage Max FaroArm) (McPherson et al., 2005) was used to take a single point (defined in all 3 axes x, y, z) on each of the 3 fiducial markers and 20 on the cup rim (Figure 1). This process was repeated 12 times by each of the two observers, for both pelvises. While the 3 fiducial marker points defined the APP, the 20 points taken on the cup rim defined the plane of the acetabular component’s cup face, known as the ‘cup plane’. The angular relationship between these 2 planes was computed using a commercially available software. Inclination was defined as the angle between the cup plane and the transverse anatomical plane of the pelvis (orthogonal to the APP). Anteversion was defined as the angle between the cup plane and the sagittal anatomical plane of the pelvis (orthogonal to both the APP and transverse plane) (Murray, 1993; Zhang et al., 2014). The values calculated represent ‘true values’ for the 3D orientation of the cup in the pelvis using the APP as the frame of reference.

### 2.2 CT imaging of the implanted cups

The pelvic model with the implanted acetabular cup and its corresponding femoral component was scanned using a 16-slice CT scanner (Siemens SOMATOM Emotion eco 16-slice configuration) (Figure 1). The images were acquired axially using a spiral sequence and at 0.75 mm increments with a kV of 100, mAs of 100 and a pitch of 1. The pelvic construct was CT scanned in 3 supine orientations: A) parallel to the floor; B) 10° of lateral pelvic tilt; and C) 20° of anterior pelvic tilt. Following the scanning process the images were saved in Digital Imaging and Communications in Medicine (DICOM) format. These axial slices were then reconstructed in both the coronal and sagittal plane with respect to the pelvis’ position in the scanner and with 0.75 mm spacing.

### 2.3 Measurement of cup position using 3 CT methods

Measurement method 1: 3D CT measurement of cup position referenced from the APP, referred to as “3D APP CT.” The 2D unprocessed axial slices in DICOM format were reconstructed to produce a 3D model of the pelvis with the prosthetic cup. This 3D image was oriented in the APP but in this method the 3D reconstructed virtual model was directly used for the measurement. Markers were placed on the fiducials, along with 20 on the acetabular cup rim, which was clearly differentiated from the metallic femoral head. The compound angles between the APP and the plane of the face of the cup were computed to give the angles of inclination and version. The position measurements (angles of inclination and anteversion) of the component were converted from the anatomical definition to radiographic definition for the Faro arm and 3D-CT, using dedicated software. This ensured all measurements were according to the radiographic definition, for comparison (Murray, 1993).

Measurement method 2: 2D CT measurement of cup position using Robin 3D software with the dataset orientated to APP in the coronal plane. This is referred to as 2D APP referenced, “2D APP CT.” The 6 datasets were exported to a picture archiving (PACS) workstation. The 2D unprocessed axial slices in DICOM format were reconstructed using Robin 3D to produce a 3D model oriented in the APP from which axial slices again oriented to the APP were viewed. 2D reconstructed slices in all three planes, parallel and perpendicular to the APP were then generated and snapshots taken in both the coronal and axial planes at the equatorial region of the components. In the coronal view 2 points were selected, one on the most superior and the other on the most inferior edges of the acetabular component margins. The angles between 2 lines, one joining the 2 spines (ASIS) and a second joining the 2 points on the cup, the “inclination angle” were measured. In the axial view and at the cup’s equatorial region, 2 points were placed on the cup margin, one on the most anterior edge and the other on the most posterior edge. The angle between a line joining the 2 markers and a line at 90° to the horizontal represents the angle of anteversion. Measurements were made 12 times by 2 observers.

Measurement method 3: 2D CT, referenced off the position of the pelvis in the CT scanner, referred to as 2D scanner referenced (SR), “2D SR CT.” Measurements of the inclination and version angles were taken directly from the coronal and axial slices, respectively. The method for measurement used in the second technique (2D APP CT) was repeated here, on these un-oriented slices.

Statistical methods: Bland-Altman plots were used to assess the level of agreement between the CT methods and the digitising CMA. Inter- and intra-observer differences were assessed using the Intra-class correlation coefficient (ICC) tool on SPSS and it considered the effect of different CT measurement methods.

## 3 Results

### 3.1 CT agreement with true CMA values

Table 1 summarizes the mean difference in inclination and version angles between the digitising arm and the 3 CT-based methods, for both pelvic models. These were positioned in a CT scanner for imaging in 3 different positions (A, B, C). All possible combinations of observers, pelvises, pelvic orientations, imaging methods and number of repeats produced 432 datapoints for the angles of inclination and version. This includes 2 observers, 2 pelvises, 3 orientations, 3 methods and 12 repeats. Table 1 reveals that 2D SR CT yields values up to 34° different to the true value and is thus a less accurate method as this is very poor agreement.

**TABLE 1:** Absolute mean (+/-2SD) difference between 12-paired coordinate measuring arm and CT measurements from observer 1 for both pelvises. 3 CT methods were used (3D APP CT, 2D APP CT and 2D SR CT), each with the pelvis in 3 different orientations in the CT scanner. Measurements were made of the cup in terms of inclination and version angles.

			Inclination angle (⁰)	
Pelvis	Orientation	D Arm-3D APP CT	D-Arm-2D APP CT	D Arm-2D SR CT
1	A	0.06 ± 0.07	0.1 ± 0.32	2.9 ± 1.2
1	B	0.23 ± 0.07	0.36 ± 0.20	0.12 ± 1.09
1	C	0.22 ± 0.08	0.17 ± 0.31	6.04 ± 2.48

			Version angle (⁰)	
Pelvis	Orientation	D Arm-3D APP CT	D-Arm-2D APP CT	D Arm-2D SR CT
1	A	0.36 ± 0.14	3.38 ± 0.36	12.9 ± 1.9
1	B	0.23 ± 0.10	3.00+ /-0.30	9.60 ± 0.84
1	C	0.41 ± 0.11	2.77 ± 0.38	19.9 ± 1.08

			Inclination angle (⁰)	
Pelvis	Orientation	D Arm-3D APP CT	D-Arm-2D APP CT	D Arm-2D SR CT
2	A	0.22 ± 0.07	0.20 ± 0.16	4.23 ± 0.88
2	B	0.24 ± 0.07	0.36 ± 0.09	8.31 ± 1.47
2	C	0.31 ± 0.06	0.33 ± 0.16	2.06 ± 1.50

			Version angle (⁰)	
Pelvis	Orientation	D Arm-3D APP CT	D-Arm-2D APP CT	D Arm-2D SR CT
2	A	0.13 ± 0.08	14.7 ± 0.29	14.8 ± 0.64
2	B	0.28 ± 0.08	14.1 ± 0.37	18.6 ± 0.66
2	C	0.13 ± 0.07	14.1 ± 0.28	34.3 ± 0.71

Supplementary Table S1 shows the average inclination and version measurements taken to define the orientation of the acetabular cup. The differences in the measurements between the digitising arm and the 3 CT methods is evident; the values obtained from 3D APP CT closely follows the Faro arm, whereas 2D SR CT reveals the largest mismatch particularly for the version angles. This is true for both pelvises.

**FIGURE 2:**
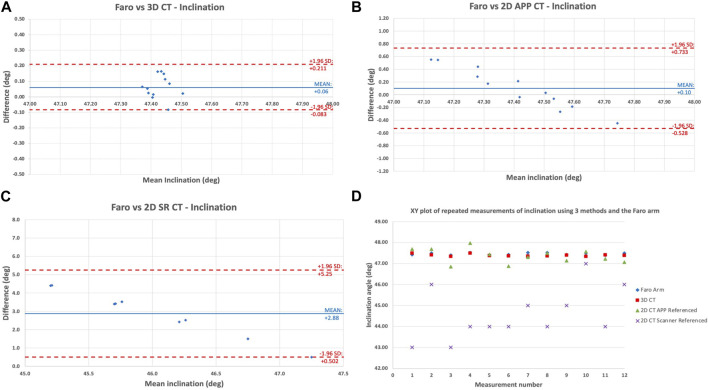
Bland-Altman plot showing the level of agreement/disagreement between the digitising arm inclination measurements and **(A)** 3D APP CT; **(B)** 2D APP CT; **(C)** 2D SR CT; **(D)** XY scatter plot of the 12 repeated measurements of inclination angles according to each of the 3 imaging methods and the Faro digitising arm for pelvis 1A by observer 1.

**FIGURE 3:**
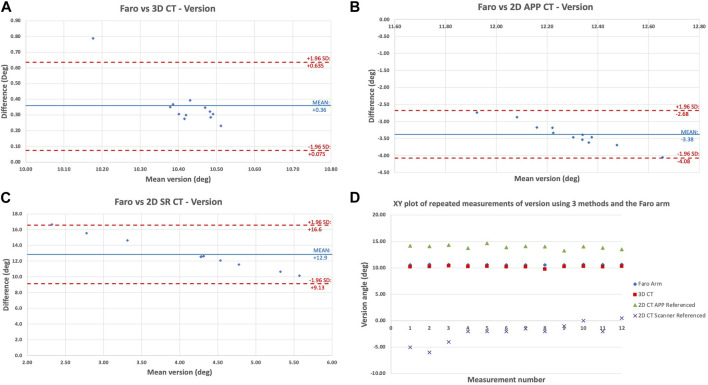
Bland-Altman plot showing the level of agreement/disagreement between the digitising arm version measurements and **(A)** 3D APP CT; **(B)** 2D APP CT; **(C)** 2D SR CT; **(D)** XY scatter plot of the 12 repeated measurements of version angles according to each of the 3 imaging methods and the Faro digitising arm for pelvis 1A by observer 1.

Bland-Altman and XY plots (Figures 2, 3) were used to assess the difference between the three CT methods and the CMA. Figure 2 compares the digitising arm values to the 3 different CT methods in terms of the measured cup inclination angle. The plots quantify the level of agreement/disagreement between the true position and the position measured using the 3 CT techniques. The solid blue line represents the mean difference, whereas the dashed lines represent the 95% limits of agreement (mean difference+/-1.96 SD). 3D APP CT and 2D APP CT showed good agreement with the Faro arm whereas the 2D SR CT measurements showed very poor agreement.

Figure 3 shows the same comparison of methods but for the cup version angle. This showed the same pattern of agreement/disagreement, however the spread of error recorded was greater than for inclination angle. Overall, there is high validity of the 3D APP CT method with decreasing validity from 3D APP CT to 2D APP CT to 2D SR CT. Once again, for version, the 3D APP CT and 2D APP CT measurements are close to the true (Faro arm) value, but less accurate compared with inclination. Figures 2, 3 are a true representation of the patterns seen for both pelvises in all three positions. The 2D SR CT measurements consistently produced the greatest error, disregarding the outlier shown for pelvis 1 in position B in Table 1. The XY plot (Figures 2, 3D) also shows that the discrepancy between the digitising arm value was lowest for 3D APP CT and highest for 2D SR CT.

**FIGURE 4:**
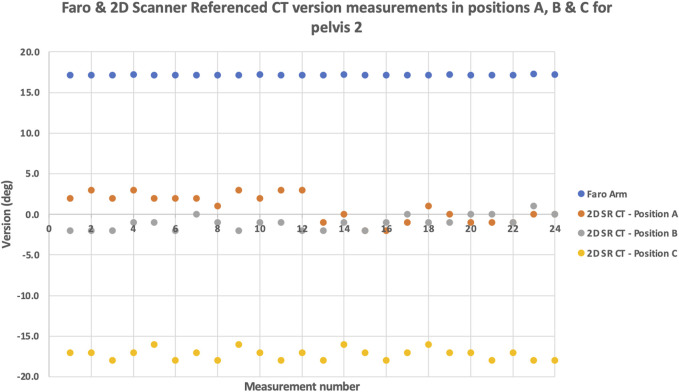
The effect of pelvic orientation: XY scatter plot of the repeated version angle measurements using the digitising arm and 2D SR CT for pelvis 2. The measurements were obtained at different pelvic orientations **(A,B,C)**.

Figure 4 shows the effect of the different pelvic orientations (A, B, C) on cup version. The method chosen for display was 2D SR CT for pelvis 2, as this showed the greatest level of disagreement between the orientations. For position C all measurements over 30° different to the digitising arm value. This result is noteworthy yet not surprising, particularly for radiographers, as it confirms that the position of the patient within the scanner will affect the measurements made. So, the 20° anterior pelvic tilt resulted in even greater disagreement between the 2D measurement and the true digitising arm position measurements.

### 3.2 ICC analysis

To measure inter-observer agreement/disagreement, intra-class correlation coefficients (ICC) of the measurements (*n* = 216) taken from both observers were calculated. ICC analysis between the observers showed excellent agreement (>0.9) for both inclination and version values. ICC analysis also showed good agreement for both observers between the CMA and 3D-CT but poor agreement between the CMA and 2D-CT methods. Irrespective of the observer, the ICC correlation between the Faro arm and 3D-CT was above 0.9 for inclination and version. The correlation between the Faro arm and 2D APP CT showed moderate agreement (0.5< ICC <0.75) for version but excellent agreement for inclination. This reaffirms that measurement of the version angle is a more difficult task to undertake, compared with inclination. Finally, the ICC value for Faro vs. 2D SR CT was below 0.5, denoting poor agreement.

## 4 Discussion

Our study is the first to quantify the potential errors associated with the use of 3D-CT for the measurement of cup angles of the acetabular component in metal-on-metal (MOM) hip replacement. Despite the frequent use of 3D-CT for the measurement of implant position, it is yet unknown how accurately this method can do so. There are several investigations in the literature which compare inclination and version measurements between different imaging methods, including 3D-CT and 2D radiographs (Davda et al., 2015), and also 3D hipEOS and 3D-CT (Anderson et al., 2022). Findings from these studies have proven 2D measurements to be less reliable, particularly when measuring cup version, while hipEOS can provide comparable angular measurements to 3D-CT. But these are clinical imaging studies of patients and only provide a comparison of different techniques, not the accuracy and precision of 3D-CT, which this lab-based study aimed to offer. Validation of 3D-CT is difficult to achieve directly from patients, given the invasive technique that would be required to take measurements using a sterilized digitising arm intra-operatively.

This study was designed to take account of the main variables that may affect the measurement of cup orientation. Firstly, the MOM relationship of the components blurring the boundaries between the cup and head components. Secondly, the position of the pelvis in the CT scanner and thirdly, the effect of the observer in terms of inter- and intra-observer error.

The composite sawbones used here provide a uniform test bed with physical properties similar to that of real bone (Heiner, 2008). These medical models are primarily used for the testing of prosthetic implant fixation and provide a reliable alternative to cadavers. The CT imaging protocol was developed for clinical use, the aim being to minimize radiation dose whilst maintaining adequate image quality, following the guidelines of The Ionising Radiation (Medical Exposure) Regulations 2000 (IR (ME)R) (Health Department, 2001). Many measurements (*n* = 432) were taken to analyse the effect of error from the method of measurement, the pelvic position, and the observer.

Metal artefact reduction strategies: The artefacts produced by imaging prosthetic implants are as a result of their constituent metals being high x-ray absorbers, reducing the amount of radiation energy transmitted through the implant to the detector, making valid measurement difficult (Berg et al., 2006). This obstruction of the x-ray beam results in the distortion of the images, leading to uncertainties in component position by the reading clinician.

The use of conventional imaging protocols produces streaking artefacts from the metal implant. To minimize the effect of these metal artefacts on the images we used both a specific image acquisition protocol and software solutions. Our imaging sequence made use of an extended Hounsfield scale (Hounsfield Units are the Supporting Material units for density as used in CT imaging) (Hounsfield, 1973). The software allowed visualization of the high-density profile of the metal component with a minimum of streaking artefacts: these images can be viewed at chosen Hounsfield thresholds. A Hounsfield threshold of 6,000 clearly visualizes cobalt-chromium. Whilst there is no single best strategy to remove these artefacts, we utilized software strategies that allowed the extension of the Hounsfield scale to define the component edges more sharply (Itokawa et al., 2008; Rinkel et al., 2008). Although some studies have shown that by manipulating the imaging acquisition parameters - mainly increasing the scanning kV - the volume of these artefacts can be reduced but not eliminated (Moon et al., 2008), we chose not to adopt this approach as the radiation dose to the patient is also increased as a result of this.

Component position measurement: The inclination angle was measured relative to a plane orthogonal to the APP-ordinarily a horizontal line when the patient is standing or when the observer is looking at an AP radiograph of the pelvis. The ‘radiographic inclination’ is easily approximated on a plain AP radiograph as the angle between the cup face and a horizontal line drawn between the ischial prominences or the lowest aspect of the teardrop. The anteversion angle is harder to describe and even harder to measure satisfactorily using plain radiographs. It is the angle between the cup face and a line in the mid coronal axis. CT on the other hand images a volume from which 2D slices can be viewed in almost any region and orientation. Others have shown the benefit of CT when compared to standard radiograph measurements of the acetabular cup (Snijders et al., 2019) but have not published the validation of their methods. Presumably they assumed that CT scanning is a validated imaging modality. However, the application of CT to MOM hip component measurement has not been compared against a reference standard. This issue is more relevant when new CT protocols and 3D measurement software are employed. Such techniques become more important when required to overcome the difficult situation presented by MOM hips, where the large diameter and high-density head obscures the imaging of the cup face.

The measurements made from our optimized 2D images revealed that the largest deviation from the true value (as determined by the CMA) occurred when the orientation of the pelvis was rotated with respect to the gantry of the scanner. This deviation was found to be greater when measuring the angle of anteversion. Conversely, both accuracy (deviation from the true value) and precision (repeatability) were improved when the pelvis was positioned in the supine position, parallel to the table. We recommend that attention is paid to the positioning of the patient’s pelvis in ensuring that the landmarks used to the derive APP are as parallel to the table as possible particularly when using the scanner as the frame of reference for measurement. Further improvements in accuracy and precision were achieved using 2D orientated CT (2D APP CT). The angles derived from the 3D APP referenced CT (3D APP CT) measurement method were least affected by the orientation of the pelvis.

We would like to emphasise that our protocol includes high-resolution 0.75 mm collimations to image both bone and prosthetic components and enables ‘separation’ of the edges of two metal components, the cup and head held in close proximity. The narrower slices increase the resolution of the images and are particularly useful for large MoM hips.

In method 1 (3D APP CT) we compared like with like: the ‘radiographical’ definition of inclination and version angles were used to compare the values for both the CMA and the virtual 3D model. The very small errors were probably due to the presence of the low-level metal artefacts affecting the labelling of the 20 points on the cup margin. We used high resolution CT to minimize this source of error (Lou et al., 2007). The accuracy of the calibrated digitising arm, our ‘reference standard’, used here, far outstrips the current resolution of clinical CT. In method 2 (2D APP CT) we standardized the frame of reference to the APP. The error between this method and the digitising arm may have been due to the fact that only two points were used to label the cup margin and the issues inherent in using a 2D view (i.e., was the most representative 2D “snapshot” selected?). The error between method 3 (2D SR CT) and the digitising arm was most likely due to the difference in the frame of reference.

A number of studies have shown good inter- and intra-observer agreement on measurements of inclination made on plain radiographs, i.e., precision. However, accuracy (closeness to the true value) is poor when compared to APP referenced CT (Bayraktar et al., 2017). Measurements on these plain 2D images are referenced off the position of the patient in the radiograph (Nishino et al., 2013) and are further confounded by the diverging source of x-ray. The close agreement between the observers in this study shows that our 3D APP CT method is also very precise.

Davda et al. (2015) also measured cup version and inclination of MOM hips using 3D-CT and Ein Bild Roentgen Analyse (EBRA) software (for 2D radiographic analysis). Their results revealed underestimated values of cup version using the 2D method. This is also true for the results of the present study. Supplementary Table S1 shows that the 2D SR CT version measurements are consistently lower than the reference angles. However, Davda et al. (2015) used 3D-CT as their gold standard measurement, for comparison. Equally, Ma et al. (2022) compared the orientation measurements taken using low-dose bi-planar radiographs (EOS imaging) with 3D-CT, but their results endorsed the use of EOS imaging for post-THA component orientation measurements. This study is the first to compare 3D-CT with a laboratory digitising arm, which is a gold standard for measurements in engineering.

With ongoing advancements in hip replacement surgery, numerous tools, including navigation, robotic and augmented reality systems, and PSI are offered to the orthopaedic surgeon to assist them in achieving optimal implant positioning. These will determine the performance of the prosthesis. If these instrumentations and surgical techniques are to be adopted in the operating theatre, there needs to be evidence that the orientation and position of the implant achieved with their use matches the surgical plan. This study sought to quantify the errors in the placement of the acetabular component using different measurement techniques and coordinate systems of the CT data, relative to a reference standard digitising arm. We have demonstrated that 3D-CT measurements can be used to perform post-operative radiological assessment and implant surveillance.

We acknowledge that this study has its limitations. The measurements were limited to the acetabular component only and the position of the femoral component was not measured. Thus, the errors associated with CT measurements of the femoral stem may be quantified in future research. We are aware of the better image resolution and faster acquisition time associated with a 64-slice CT scanner (hence giving more accurate results), but we scanned the pelvis using a more widely available 16-slice CT scanner to represent a worst-case scenario for the measurements taken. A more modern 64-slice scanner may be used in future studies to give even more accurate angular measurements. This study measured the accuracy of the acetabular implant performed conventionally by the surgeon. By contrast, in computer-aided surgery, the size and implant position can be pre-planned (Inoue et al., 2019). However, this study did not include this aspect.

## 5 Conclusion

In conclusion, this study validates the use of 3D-CT for the measurement of acetabular component positioning post-operatively. Although this lab-based study involved an artificial pelvic bone model, the results may be extended in the context of clinical CT images of real patients. The differences found in the measurements from the variable CT methods, necessitates this paper. This is because the measurements taken from the same CT scan before and after specialized 3D-rendering software is used to manipulate the image (by orientating the APP to the coronal), are shown to differ. This emphasizes the need for additional software which is not available on the CT console or most PACS systems.

Here, the accurate measurement of inclination and anteversion in the context of an unknown pelvic orientation presents a fundamental 3-dimensional challenge. The challenge is increased in the context of large diameter MOM hips. In this study we have demonstrated and validated the use of 3D-CT to measure the anatomical angles of inclination and version (later converted into the radiographic definitions of the angles). The ability to standardize the measurements of cup orientation to the accepted frame of reference (APP) in three dimensions will allow surgeons and researchers to more accurately study and plan surgery. This will further the study of the relationship between component position and outcome, such as the investigation of painful and poorly functioning prosthetic hips.

Many studies have used 2D-CT measurement methods and this work has shown that 3D-CT methods offer superior accuracy and therefore we recommend the use of 3D-rendering software solutions. Our CT protocol has significant clinical improvement over others because it uses a low radiation dose and minimizes metal artefact. Although the study was performed on MOM prosthesis, we are currently using the method here developed on metal-on-polyethylene and ceramic-on-ceramic prostheses where the challenges in identifying the component boundaries are not as great. Our study is very pertinent because we are bettering our understanding in the large variability of post-operative component position and its effect on function and failure rates. With an ever-increasing demand for high performance hips from the young, more active and an ageing population, the orthopaedic surgeon of today needs additional tools in his armamentarium to further study the function and longevity (Deep et al., 2017) of hip replacement surgery.

## Data Availability

The datasets presented in this article are not readily available because none. Requests to access the datasets should be directed to Angelika.ramesh.18@ucl.ac.uk.

## References

[B1] Anderson C. G. Brilliant Z. R. Jang S. J. Sokrab R. Mayman D. J. Vigdorchik J. M. (2022). Validating the use of 3D biplanar radiography versus CT when measuring femoral anteversion after total hip arthroplasty. Bone. Jt. J. 104-B, 1196–1201. 10.1302/0301-620x.104b11.bjj-2022-0194.r2 36317354

[B2] Bayraktar V. Weber M. von Kunow F. Zeman F. Craiovan B. Renkawitz T. (2017). Accuracy of measuring acetabular cup position after total hip arthroplasty: Comparison between a radiographic planning software and three-dimensional computed tomography. Int. Orthop. 41, 731–738. 10.1007/s00264-016-3240-1 27277948

[B3] Berg V. B. Malghem J. Maldague B. Lecouvet F. (2006). Multi-detector CT imaging in the postoperative orthopedic patient with metal hardware. Eur. J. Radiol. 60, 470–479. 10.1016/j.ejrad.2006.08.008 17079106

[B4] Brownlie C. A. Evans R. Morrison D. Hayes A. Song S. Kuster M. S. (2020). Improved accuracy of CT based measurements for anterior prominence of acetabular prosthesis using a novel protocol based on Anatomical Landmarks. Orthop. Traumatol. Surg. Res. 106, 563–568. 10.1016/j.otsr.2019.10.019 31959362

[B5] Davda K. Smyth N. Cobb J. P. Hart A. J. (2015). 2D measurements of cup orientation are less reliable than 3D measurements. Acta Orthop. 86, 485–490. 10.3109/17453674.2015.1017791 25674698 PMC4513605

[B6] Deep K. Shankar S. Mahendra A. (2017). Computer assisted navigation in total knee and hip arthroplasty. SICOT-J 50, 50–56. 10.1051/sicotj/2017034 PMC5532908 28752819

[B7] Elson L. Dounchis J. Illgen R. Marchand R. C. Padgett D. E. Bragdon C. R. (2015). Precision of acetabular cup placement in robotic integrated total hip arthroplasty. Hip Int. 25, 531–536. 10.5301/hipint.5000289 26391264

[B8] Fontalis A. Epinette J-A. Thaler M. Zagra L. Khanduja V. Haddad F. S. (2021). Advances and innovations in total hip arthroplasty. SICOT-J 7, 26–10. 10.1051/sicotj/2021025 33843582 PMC8040589

[B9] Health Department (2001). The ionising radiation (medical exposure) Regulations 2000: Programme of inspections (S.I. 2000/1059). Scotland: Health Department.

[B10] Heiner A. D. (2008). Structural properties of fourth-generation composite femurs and tibias. J. Biomech. 41, 3282–3284. 10.1016/j.jbiomech.2008.08.013 18829031

[B11] Henckel J. Holme T. J. Radford W. Skinner J. A. Hart A. J. (2018). 3D-printed patient-specific guides for hip arthroplasty. J. Am. Acad. Orthop. Surg. 26, 342–348. 10.5435/jaaos-d-16-00719 30052547

[B12] Hounsfield G. N. (1973). Computerized transverse axial scanning (tomography): Part 1. Description of system. Br. J. Radiol. 46, 1016–1022. 10.1259/0007-1285-46-552-1016 4757352

[B13] Inoue D. Kabata T. Kimura H. Tsuchiya H. (2019). A prospective clinical trial to assess the accuracy of an MRI-based patient-specific acetabular instrument guide in total hip arthroplasty. Eur. J. Orthop. Surg. Traumatol. 29, 65–71. 10.1007/s00590-018-2279-7 30132077

[B14] Itokawa H. Hiraide T. Moriya M. Fujimoto M. Nagashima G. Suzuki R. (2008). The influence on the images of computed tomography caused by the use of artificial cranial reconstructive materials. No Shinkei Geka 36, 607–614. 18634403

[B15] Kaiser M. Renkawitz T. Benditz A. König M. Thieme M. Weber M. (2021). Pelvic tilt impacts cup orientation on CT: How accurate is the gold standard? Acta Radiol. 63, 698–705. 10.1177/02841851211009466 33982602

[B16] Krämer M. Kahrs L. A. Friese K-I. von Falck C. Hurschler C. (2018). Inter- and intra-operator reliability in patient-specific template positioning for total hip arthroplasty. A cadaver study. Int. J. Med. Robot. 14, 18877–e1896. 10.1002/rcs.1887 29336121

[B17] Lou L. Lagravere M. O. Compton S. Major P. W. Flores-Mir C. (2007). Accuracy of measurements and reliability of landmark identification with computed tomography (CT) techniques in the maxillofacial area: A systematic review. Oral Surg. Oral Med. Oral Pathol. Oral Radiol. Endod. 104, 402–411. 10.1016/j.tripleo.2006.07.015 17709072

[B18] Ma Z. Tang H. Zhou Y. Wang S. Yang D. Guo S. (2022). Assessing component orientation of total hip arthroplasty using the low-dose bi-planar radiographs. BMC Musculoskelet. Disord. 23, 886–889. 10.1186/s12891-022-05835-3 36154920 PMC9511787

[B19] McPherson A. Karrholm J. Pinskerova V. Sosna A. Martelli S. (2005). Imaging knee position using MRI, RSA/CT and 3D digitisation. J. Biomech. 38, 263–268. 10.1016/j.jbiomech.2004.02.007 15598452

[B20] Moon S. G. Hong S. H. Choi J. Y. Jun W. S. Kang H. G. Kim H. S. (2008). Metal artifact reduction by the alteration of technical factors in multidetector computed tomography: A 3-dimensional quantitative assessment. J. Comput. Assist. Tomogr. 32, 630–633. 10.1097/rct.0b013e3181568b27 18664853

[B21] Murray D. W. (1993). The definition and measurement of acetabular orientation. J. Bone Jt. Surg. Br. 75, 228–232. 10.1302/0301-620x.75b2.8444942 8444942

[B22] Musielak B. Kubicka A. M. Rychlik M. Czubak J. Czwojdziński A. Grzegorzewski A. (2019). Variation in pelvic shape and size in eastern European males: A computed tomography comparative study. PeerJ 7, 64333–e6524. 10.7717/peerj.6433 PMC6387581 30809442

[B23] Nishino H. Nakamura S. Arai N. Matsushita T. (2013). Accuracy and precision of version angle measurements of the acetabular component after total hip arthroplasty. J. Arthroplasty 9, 1644–1647. 10.1016/j.arth.2013.02.014 23566697

[B24] Rinkel J. Dillon W. P. Funk T. Gould R. Prevrhal S. (2008). Computed tomographic metal artifact reduction for the detection and quantitation of small features near large metallic implants: A comparison of published methods. J. Comput. Assist. Tomogr. 32, 621–629. 10.1097/rct.0b013e318149e215 18664852

[B25] Snijders T. E. Schlösser T. P. van Gaalen S. M. Castelein R. M. Weinans H. de Gast A. (2019). Non-equivalent results from different anteversion measurements methods for the evaluation of the acetabular cup orientation in total hip arthroplasty. Orthop. Surg. 11, 241–247. 10.1111/os.12445 30932341 PMC6594505

[B26] Spencer-Gardner L. Pierrepont J. Topham M. Baré J. McMahon S. Shimmin A. J. (2016). Patient-specific instrumentation improves the accuracy of acetabular component placement in total hip arthroplasty. Bone. Jt. J. 98, 1342–1346. 10.1302/0301-620x.98b10.37808 27694587

[B27] Zhang J. Wang L. Mao Y. Li H. Ding H. Zhu Z. (2014). The use of combined anteversion in total hip arthroplasty for patients with developmental dysplasia of the hip. J. Arthroplasty 29, 621–625. 10.1016/j.arth.2013.08.004 24029717

