# Supplementary material for: The accuracy and precision of acetabular implant measurements from CT imaging
Source: Front Bioeng Biotechnol. 2023 Apr 28;11:1150061. doi: 10.3389/fbioe.2023.1150061 (PMC10175694; doi:10.3389/fbioe.2023.1150061)
Supplement: Supplementary file 1 [file Table1.docx]

Supplementary Material

The accuracy and precision of acetabular implant measurements from CT imaging

Johann Henckel†, Angelika Ramesh^*^†, Harry Hothi, Robin Richards, Anna Di Laura, Alister Hart

*** Correspondence:** Corresponding Author: Angelika Ramesh, angelika.ramesh.18@ucl.ac.uk

# Supplementary Figures and Tables

Table S1. The average (+/- SD) inclination and anteversion measurements taken for the acetabular component from the CMA and CT scanner for both pelvises in each of the 3 orientations of the pelvis within the scanner. 3 CT methods were used (3D APP CT, 2D APP CT and 2D SR CT).

| Pelvis | Orientation | Digitising arm | 3D APP CT | 2D APP CT | 2D SR CT |
| --- | --- | --- | --- | --- | --- |
|  |  | Inclination (°) | | | |
| 1 | A | 47.47 ± 0.05 | 47.45 ± 0.074 | 47.50 ± 0.53 | 40.96 ± 3.94 |
| 1 | B | N/A* | 47.34 ± 0.13 | 47.27 ± 0.43 | 41.71 ± 6.10 |
| 1 | C | N/A* | 47.30 ± 0.09 | 47.04 ± 0.62 | 42.29 ± 2.12 |
|  |  | Version (°) | | | |
| Pelvis | Orientation | Digitising arm | 3D APP CT | 2D APP CT | 2D SR CT |
| 1 | A | 10.6 ± 0.04 | 10.23 ± 0.11 | 12.96 ± 1.18 | 3.5 ± 6.11 |
| 1 | B | N/A* | 10.36 ± 0.06 | 12.17 ± 1.50 | 2.5 ± 1.79 |
| 1 | C | N/A* | 10.28 ± 0.13 | 12.53 ± 1.11 | -9 ± 0.93 |
|  |  | Inclination (°) | | | |
| Pelvis | Orientation | Digitising arm | 3D APP CT | 2D APP CT | 2D SR CT |
| 2 | A | 30.49 ± 0.04 | 30.27 ± 0.07 | 30.33 ± 0.46 | 29.29 ± 3.25 |
| 2 | B | N/A* | 30.25 ± 0.05 | 30.33 ± 0.38 | 23.38 ± 1.74 |
| 2 | C | N/A* | 30.20 ± 0.06 | 30.16 ± 0.68 | 28.46 ± 1.32 |
|  |  | Version (°) | | | |
| Pelvis | Orientation | Digitising arm | 3D APP CT | 2D APP CT | 2D SR CT |
| 2 | A | 17.17 ± 0.04 | 17.26 ± 0.06 | 31.66 ± 0.91 | 0.83 ± 1.71 |
| 2 | B | N/A* | 17.41 ± 0.06 | 31.75 ± 0.66 | -1.04 ± 0.86 |
| 2 | C | N/A* | 17.26 ± 0.06 | 32.25 ± 1.31 | - 17.21 ± 0.72 |

*Not applicable as the Faro arm measurements were taken with the pelvis in a single position.
